# C4d Deposition in Fetal Vessels of the Placenta in Neonatal Lupus Syndrome

**DOI:** 10.1155/2019/5863476

**Published:** 2019-03-25

**Authors:** Yuichiro Sato, Tomoko Yamaguchi, Jyunsuke Muraoka, Hajime Taniguchi, Atsushi Kisanuki, Kazunari Maekawa, Atushi Yamashita, Murasaki Aman, Yuki Kodama, Hiroshi Sameshima, Yujiro Asada

**Affiliations:** ^1^Department of Diagnostic Pathology, Faculty of Medicine, Miyazaki University Hospital, University of Miyazaki, 5200 Kihara, Kiyotake, Miyazaki 889-1692, Japan; ^2^Department of Obstetrics and Gynecology, Miyazaki Prefectural Nichinan Hospital, 1-9-5, Kiyama, Nichinan, Miyazaki 887-0013, Japan; ^3^Department of Diagnostic Pathology, Miyazaki Prefectural Nichinan Hospital, 1-9-5, Kiyama, Nichinan, Miyazaki 887-0013, Japan; ^4^Department of Pathology, Faculty of Medicine, University of Miyazaki, 5200 Kihara, Kiyotake, Miyazaki 889-1692, Japan; ^5^Department of Obstetrics and Gynecology, Faculty of Medicine, University of Miyazaki, 5200 Kihara, Kiyotake, Miyazaki 889-1692, Japan

## Abstract

Neonatal lupus syndrome (NLS) is a rare, passively acquired autoimmune syndrome caused by maternal autoantibodies. We describe a case of a newborn with NLS and the accompanying placental findings. A female neonate was born by emergency cesarean delivery due to non-reassuring fetal status at 35 weeks and 3 days. This neonate had congenital erythematous and scar lesions on the face, back, and upper and lower extremities. Maternal and fetal anti-SSA and SSB antibodies were elevated and this baby was diagnosed as NLS. Histologically, the chorionic villi demonstrated capillary shrinkage. An immunohistochemical study revealed complement deposition (C4d) in the capillaries of the villi and umbilical vessels. Our findings suggest that maternal autoantibodies affect the inflammatory response of the fetus through the placenta and that C4d deposition may be useful for diagnosing NLS.

## 1. Introduction

Neonatal lupus syndrome (NLS) is an acquired autoimmune disease caused by the transplacental passage of maternal anti-SSA/Ro and/or anti-SSB/La antibodies [1, 2]. However, the pathological findings in the placenta of neonates with NLS have not been documented.

Complement deposition (C4d) is a split product of C4b and C4d deposition in vascular endothelial cells with organ rejection, and it is generally considered evidence for classical complement pathway activity [3]. Recently, C4d deposition in the placenta was reported for systemic lupus erythematosus (SLE), preeclampsia, and miscarriage [4, 5]; however, the histological findings of NLS have not been documented. We describe a case of a newborn with NLS and the accompanying placental findings of C4d deposition.

## 2. Case Presentation

**Figure 1:**
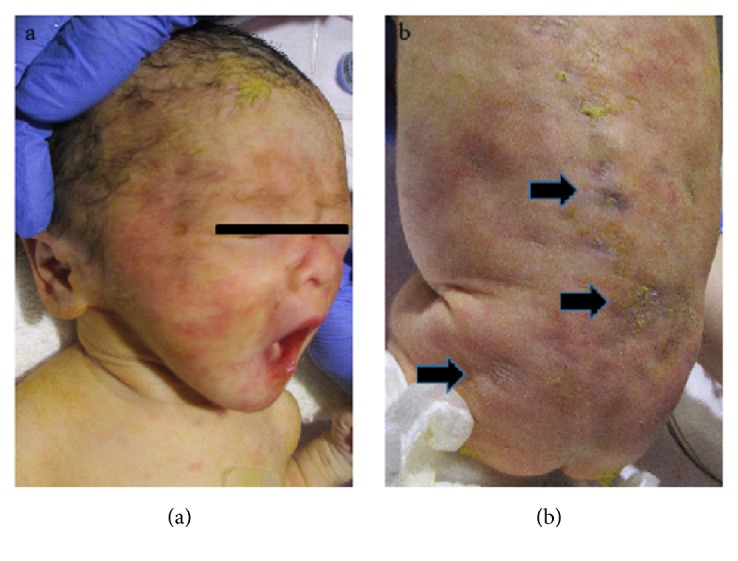
Skin lesions. (a) Skin rash with annular erythematous lesions on the face. (b) Annular, erythematous, scaly, atrophic patches on the trunk and back (arrows).

A 31-year-old woman (gravida 5, para 2) was referred to our hospital due to labor pain. She was suspected to have SLE because of facial erythema at age 29 years, but clinical and serological findings failed to satisfy the diagnosis criteria for SLE. She gave birth to two boys without any problems at age 23 years (birth weight, 2822 g) and 26 years (birth weight, 2946 g). The fetal heart monitor showed non-reassuring fetal status. A female neonate was born by emergency cesarean delivery at 35 weeks and 3 days. The neonate weight was 1,909 g. She had fetal growth restriction (FGR) and Apgar scores of 5 and 7 (1 minute and 5 minutes). Her skin was pale. She had congenital erythematous and scar lesions on the face, back, and upper and lower extremities (Figure 1). An examination revealed a slight elevation in hepatic transaminases, thrombocytopenia, and mild cardiac failure. No heart blockage was detected. The serological examination of the neonate showed elevated anti-SSA/Ro (281 U/mL) and anti-SSB/La antibodies (≧1000 U/mL). Other antibodies were normal range (anti-DNA antibody ≦2.0 IU/mL, anti-RNP antibody <2.0 U/mL), and complements were not reduced (C3; 92 mg/dl, C4; 22 mg/dl). The serological examination of the mother also showed elevation of these antibodies, and a histological examination demonstrated lymphocytic infiltration of the minor salivary gland; therefore, a diagnosis of Sjögren syndrome was made. The symptoms of the neonate had almost resolved by 7 months of age.

**Figure 2:**
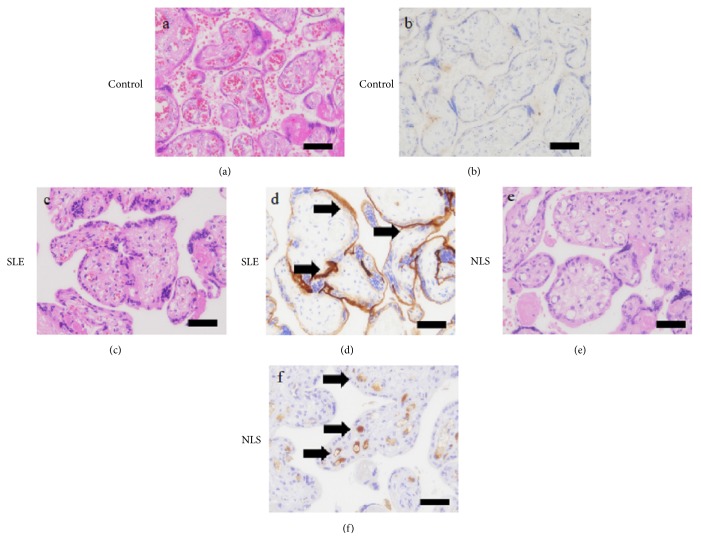
Histological findings from placentas obtained from the control pregnancy (28-year-old mother at 35 weeks of gestation, (a, b)), systemic lupus erythematous (SLE) pregnancy (40-year-old mother at 30 weeks of gestation, (c, d)), and neonatal lupus syndrome pregnancy (31-year-old mother at 35 weeks of gestation, (e, f)). (a, c, e) Representative images of tissue sections stained with hematoxylin and eosin (HE). (b, d, f) Representative anti-C4d immunostaining images (arrows). Scale bar = 50 *μ*m.

The placenta was 17 x 13.5 x 2 cm and weighed 285 g. The amnion color was green, and the cut surface showed anemia. Histological examination revealed collapsed capillaries in the terminal villi (Figure 2(e)). No apparent inflammatory cells or thrombus formation was found in the fetal vessels. Partial focal maternal vessel thrombosis was noted, but no apparent infarction was seen. A meconium stain with amnion degeneration was observed in the amnion. Focal maternal thrombosis and increased syncytial knots were also present.

Immunohistostaining of a normal placenta (Figure 2(b)), placenta with maternal SLE (Figure 2(d)), and the placenta involved in this case (Figure 2(f)) was performed using C4d (ABGENT, San Diego, CA, USA). In the normal placenta, focal or weak C4d deposits were present at the syncytiotrophoblasts, but not in the lumen of the vessels (Figure 2(b)). C4d deposits were strongly observed at the syncytiotrophoblasts in SLE cases (Figure 2(d)). The placenta with NLS showed C4d deposition in the lumen of the capillaries of the terminal villi (Figure 2(f)). C4d deposition was also seen in the stem vessels, chorionic vessels, and umbilical vessels in this case. C4d deposition was not detected in the maternal vessels.

## 3. Discussion

We found C4d depositions in the fetal endothelial cells of the NLS placenta. C4d is commonly referred to as the “footprint” of antibody-mediated tissue injury [6]. This report demonstrated the direct findings of antibody-mediated tissue injury by examining the placenta. Capillary collapse was also detected in the placenta, but apparent thrombus formation, massive inflammatory cell infiltration, and apoptotic cells were not found with hematoxylin and eosin (HE) staining. We found C4d deposition in fetal vessels in the placenta, but not in the maternal vessels in the decidua. Our findings suggest that maternal antibody reactions across the placenta induce fetal organ injury but do not attack the placenta or maternal vessels. We hypothesized that complement is activated by maternal antibodies in the fetal vessels, through the classical pathway, cleaved C4d bounds the surface of fetal endothelium.

Even though it is an inactive split product of the complement cascade, C4d is a biomarker. Covalently bound C4d anchors tightly to the tissue; therefore, it serves as a footprint of antibody-mediated tissue injury [6]. C4d deposits are now considered the gold standard for diagnosing renal graft acute humoral resection [3]. Furthermore, several groups have shown that C4d deposition in capillaries is a reliable and specific marker of antibody-mediated injury in other transplanted solid organs that are rejected [7, 8]. Interestingly, placental C4d detected in the majority of SLE and anti-phospholipid syndrome cases has a diffuse staining pattern at the syncytiotrophoblasts [4]. We have found C4d deposition in the lumen of placental fetal vessels; however, that deposition had patterns that were different from those of our case. We studied C4d deposition in normal placenta and SLE cases, but we could not detect the capillary deposition pattern in other cases. We speculate that antibodies in SLE or anti-phospholipid syndrome are activated by maternal inflammatory cells or cytokines in the intervillous space, and this induces the C4d binding at the syncytiotrophoblasts. In NLS, these antibodies are silent in the intervillous space, and then these become activated complement cascade in the fetal circulation. However, further examinations are needed because C4d deposits may be useful markers of NLS.

Wisuthsarewong et al. [9] documented that 29.4% of NLS cases involved FGR. Our case also involved a neonate with FGR and a small placenta. In addition, histological findings revealed maternal vessel thrombosis. Maternal vasculopathy, including maternal vessel thrombosis, is associated with FGR. Several authors showed that maternal vasculopathy is related to maternal hypertension, but the association between maternal vasculopathy and collagen disease is unclear [10]. In our case, the mother had no history of hypertension; in addition, C4d deposits were not detected in the maternal vessels. Revaux et al. [11] reported that FGR and chronic intervillositis were associated with antiphospholipid syndrome and other autoimmune diseases; however, we could not detect chronic intervillositis or massive fibrin deposition. We considered that the small placenta and maternal vasculopathy were associated with FGR in this case.

NLS is a rare neonatal immune-mediated disease. Wisuthsarewong et al. [9] reported that the incidence of NLS is approximately 1 in 12,500 to 20,000 live births, and that it is slightly higher for girls and premature newborns. However, the true incidence of NLS has not been determined because of the high rate of underdiagnosed cases. Approximately 40-60% of mothers are asymptomatic when newborns are diagnosed with NLS [1, 2, 9]. Some mothers may have SLE, Sjögren syndrome, rheumatoid arthritis, or another undifferentiated autoimmune disease. Zuppa et al. [12] documented that the most frequent maternal autoimmune disease to accompany NLS was SLE (25/50); in their study, 15 mothers had primary Sjögren syndrome and 5 had SLE combined with Sjögren syndrome. In our case, the newborn's diagnosis was made based on neonatal skin findings and serum antibody levels of the newborn and the mother. The mother was suspected to have SLE 3 years before this pregnancy; after delivery, she was diagnosed with Sjögren syndrome.

The most common clinical manifestations of NLS are dermatologic, cardiac, hepatobiliary, and hematologic abnormalities [1, 2, 12]. Dermatologic lesions may be present at birth, but they often appear within the first few weeks of life. Annular erythematous or polycystic plaque with or without scarring characterizes NLS and appears predominantly on the scalp, neck, or face. Periorbital erythema, referred to as “raccoon eye” or “owl eye,” is a common characteristic. Our case also demonstrated characteristic dermatitis on the face, back, and upper and lower extremities, and the physicians suspected NLS with cutaneous lesions. The outcome of NLS is usually good when only skin lesions are present. Cardiac abnormalities, especially third-degree heart blockage, can be the most serious consequences of NLS. Li et al. [13] recently suggested that the frequency of congenital heart blockage was significantly lower for Asians than for Caucasians. Our case involved non-reassuring fetal status detected by the fetal heart monitor, but the newborn had no heart blockage. Her major symptoms were cutaneous lesions, and these have almost resolved.

## 4. Conclusion

We encountered a case of NLS and found C4d deposition in the placental capillaries and umbilical vessels. This is the first report of placental findings of NLS. These findings support the hypothesis that maternal antibodies affect the fetus through the placenta.
